# Gene duplication is the primary driver of intraspecific genomic divergence in coral algal symbionts

**DOI:** 10.1098/rsob.230182

**Published:** 2023-09-27

**Authors:** Sarah Shah, Katherine E. Dougan, Yibi Chen, Debashish Bhattacharya, Cheong Xin Chan

**Affiliations:** ^1^ School of Chemistry and Molecular Biosciences, and Australian Centre for Ecogenomics, The University of Queensland, Brisbane, 4072 Queensland, Australia; ^2^ Department of Biochemistry and Microbiology, Rutgers University, New Brunswick, NJ 08901, USA

**Keywords:** Symbiodiniaceae, coral symbionts, microalgae, dinoflagellates, gene duplication, genome evolution

## Abstract

Dinoflagellates in the order Suessiales include the family Symbiodiniaceae, which have essential roles as photosymbionts in corals, and their cold-adapted sister group, *Polarella glacialis*. These diverse taxa exhibit extensive genomic divergence, although their genomes are relatively small (haploid size < 3 Gbp) when compared with most other free-living dinoflagellates. Different strains of Symbiodiniaceae form symbiosis with distinct hosts and exhibit different regimes of gene expression, but intraspecific whole-genome divergence is poorly understood. Focusing on three Symbiodiniaceae species (the free-living *Effrenium voratum* and the symbiotic *Symbiodinium microadriaticum* and *Durusdinium trenchii*) and the free-living outgroup *P. glacialis*, for which whole-genome data from multiple isolates are available, we assessed intraspecific genomic divergence with respect to sequence and structure. Our analysis, based on alignment and alignment-free methods, revealed a greater extent of intraspecific sequence divergence in Symbiodiniaceae than in *P. glacialis*. Our results underscore the role of gene duplication in generating functional innovation, with a greater prevalence of tandemly duplicated single-exon genes observed in the genomes of free-living species than in symbionts. These results demonstrate the remarkable intraspecific genomic divergence in dinoflagellates under the constraint of reduced genome sizes, shaped by genetic duplications and symbiogenesis events during the diversification of Symbiodiniaceae.

## Graphical abstract

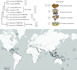

## 1. Introduction

Dinoflagellate microalgae in the order Suessiales include the family Symbiodiniaceae, which predominantly comprise symbiotic lineages essential to coral reefs. Symbiodiniaceae taxa, collectively, exhibit a broad spectrum of symbiotic associations (i.e. facultative) and variable degrees of host specificity (i.e. host-specialist versus host-generalist), although some are described as free-living [1,2]. A comparative analysis of whole-genome sequences from 15 taxa revealed extensive sequence and structural divergence among Symbiodiniaceae taxa, which was more prevalent in isolates of the symbiotic species, *Symbiodinium microadriaticum* [3]. This result was supported by a metagenomics survey of single-nucleotide polymorphisms in the genomes of symbiotic *Symbiodinium 'fitti'* from different coral taxa and biogeographic origins, revealing intraspecific (i.e. within-species) sequence divergence correlated with the coral host [4].

A recent comparative analysis incorporating genomes from three isolates of the obligate, free-living species *E. voratum* identified genome features of the putative free-living ancestor of Symbiodiniaceae [5]. These features include longer introns, more-extensive RNA editing, less pseudogenization, and, perhaps most surprisingly, similar genome sizes when compared to symbiotic counterparts. The genome size of *E. voratum* suggests that genome reduction (to haploid genome size < 3 Gbp) occurred in symbiodiniacean dinoflagellates before diversification of the order Suessiales [5]. These results elucidate the role of a symbiotic lifestyle in shaping intraspecific genomic divergence and the evolution of these taxa. Intragenomic variation of the ITS2 phylogenetic marker sequences is known among Symbiodiniaceae taxa [6,7]. However, intraspecific whole-genome divergence in these taxa relative to symbiotic versus free-living lifestyle remains poorly understood. Whole-genome data from multiple isolates of a species provide an excellent analysis platform to address this knowledge gap.

Here, we investigate intraspecific genomic divergence in four Suessiales species (of which three are Symbiodiniaceae). These taxa represent two free-living species and two symbiotic species, for which whole-genome data from multiple isolates are available. We focus specifically on sequence and structural conservation, gene family dynamics, and gene duplication, and how these features may reflect adaptation to different lifestyles.

## 2. Methods

### 2.1. Genome data

**Figure 1.**
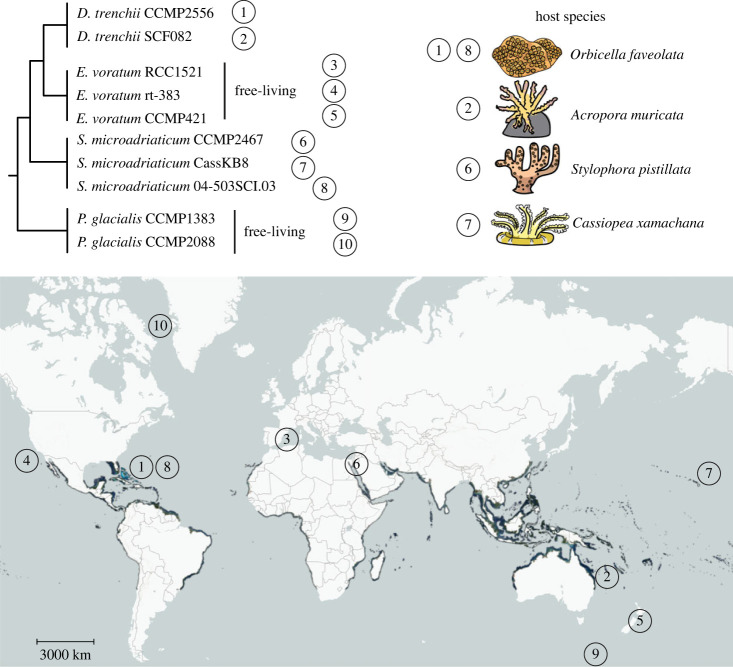
Suessiales species phylogeny inferred using LSU rDNA sequences [1], for which genome data from multiple isolates are available. Coral reef world map is based on Allen Coral Atlas [10]. Taxa not marked 'free-living' are symbiotic and their host species are represented on the top right.

To investigate patterns of intraspecific genomic divergence related to a facultative lifestyle, we focused on four Suessiales species for which multi-isolate genome data are publicly available. The two symbiodiniacean species, *S. microadriaticum* [3,8] and *Durusdinium trenchii* [9], represent independent origins of symbiogenesis (figure 1; electronic supplementary material, table S1). The remaining two are free-living species, the symbiodiniacean *E. voratum* [5] and *Polarella glacialis* that is sister to the Symbiodiniaceae in the order Suessiales [11]. The available genome data were generated from isolates collected over vast geographical areas: the thermotolerant symbiont *D. trenchii* from the Caribbean Sea and Pacific Ocean, the free-living *E. voratum* from the Mediterranean Sea and both sides of the Pacific Ocean, the symbiotic *S. microadriaticum* from the Red Sea, Pacific Ocean and the Caribbean Sea, and the psychrophilic *P. glacialis* from the Antarctic and Arctic oceans (figure 1). Collectively, these data provide the framework for interrogating intraspecific genome divergence.

### 2.2. Alignment-based assessment of genome-sequence similarity

To assess genome-sequence similarity of the four target species based on sequence alignment, we used nucmer (*—mum*) implemented in MUMmer 4.0.0beta2 [12] at minimum alignment lengths of 100 bp, 1 Kb and 10 Kb to align assembled genome sequences for every possible pair of isolates in each species. For each pairwise comparison, we calculated the percentage of aligned bases, *Q*, and overall sequence identity of aligned regions, *ID*. Maximum values of for both *Q* and *ID* at 100% indicate that two genome sequences are identical. We then used mummerplot (*-f —layout*) and dnadiff to generate figures and reports for these alignments.

### 2.3. Alignment-free assessment of genome-sequence similarity

Adopting the same approach described in Lo *et al*. [13], we calculated statistic based on shared *k*-mers for each pair of genomes, from which a distance (*d*) was derived. Briefly, Jellyfish v2.3.0 [14] was used to derive *k-*mers (at *k* = 23) from each genome assembly, from which distances were calculated using *d2ssect* (https://github.com/bakeronit/d2ssect) from all possible pairs of genomes. Following the earlier studies [5,13], core 23*-*mers among isolates of each species were identified from the extracted 23-mers, using the bash command *comm* (-12). BEDtools [15] *intersect* was used to find regions of overlap between the core *k-*mers and different genomic features.

### 2.4. Gene family evolution and introner element search

To infer homologous protein sets among isolates of a species, all protein sequences predicted from all isolates were used as input for OrthoFinder v2.5.4 [16]. The analysis was conducted at different inflation parameters (*I* = 1.5, 2.0, 4.0, 6.0, 8.0 or 10.0). From the generated homologous protein sets, the proportion of isolate-specific sets was identified. To identify introner elements (IE), we used the introner element sequences identified in Shah *et al*. [5] from eight Suessiales isolates as a reference for Pattern Locator [17] to search for inverted and direct repeat motifs within introns.

### 2.5. Identification of collinear gene blocks and types of gene duplication

To identify collinear gene blocks shared by isolates of a species, we first identified homologous protein sequences using BLASTp (e-value < 10^−5^, query or subject cover > 50%, filtered for top five hits for each query). This output was used as input for MCScanX [18] (*-b 2*) to search for collinear gene blocks between all possible pairs of isolates. For *D. trenchii,* we filtered out duplicated genes [9] from the MCScanX output by selecting gene pairs that were more similar to each other (i.e. low non-synonymous (*K_a_*) + synonymous (*K_s_*) substitution score), then chose gene blocks that still contained 5 or more genes. Gene Ontology (GO) terms were assigned to all gene sets via UniProt (version 2022_01) to GO (version December 2022) ID mapping on the UniProt website (https://www.uniprot.org/id-mapping). The *duplicate_gene_classifer* implemented in MCScanX was used to assess five distinct types of gene duplication: (a) singleton = not duplicated; (b) dispersed = duplicated with > 10 genes in between; (c) proximal = duplicated with < 10 genes in between; (d) WGD = whole or segmental genome duplication inferred by anchor genes in collinear gene blocks comprising at least 5 genes; and (e) tandem = duplicated one after the other (i.e. two or more consecutive genes on the same scaffold).

### 2.6. Analysis of tandemly duplicated genes

Tandemly duplicated (TD) genes were identified based on the results of MCScanX above. For this analysis, we focused on two best-quality (i.e. two most contiguous) genome assemblies from each species, i.e. for a total of eight genomes. For each TD block, we calculated the non-synonymous substitution rate (*K_a_*) and synonymous rate (*K_s_*) between all possible pairs of genes within the block, using the *add_ka_and_ks_to_collinearity.pl* script implemented in MCScanX [18]. The ratio *ω* was defined as *K_a_*/*K_s_*. When assessing mean *ω* for each TD block, instances of infinity values (e.g. due to *K_s_* = 0) were ignored.

## 3. Results and discussion

### 3.1. Genomes of facultative symbionts have higher sequence divergence

**Figure 2.**
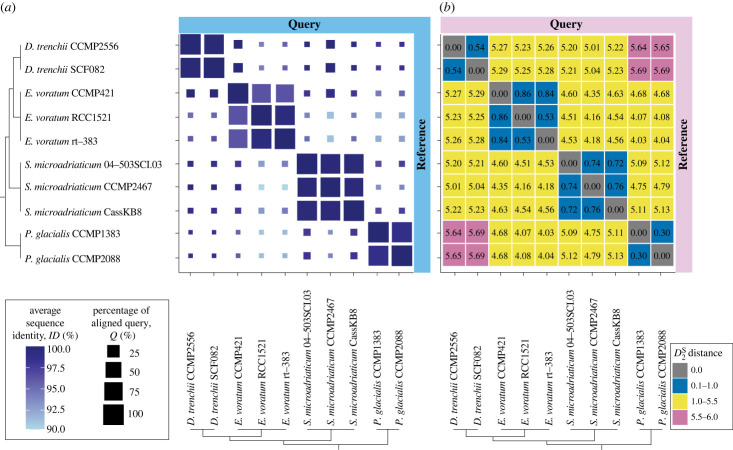
Intra- and inter-species genome sequence identity among four Suessiales species. (*a*) Alignment-based identity (minimum alignment length = 100 bp) with query genome sequences (*y*-axis) aligned to the references (*x*-axis). The colour of the squares corresponds to percentage sequence identity *ID*, and the sizes represent *Q*, the percentage of the query genome sequence aligned to the reference. (*b*) Alignment-free $D_{2}^{S}$ distances showing delineation between species (<1 in blue), family (1.0–5.5 in yellow) and the longest evolutionary distance across the order (>5.5 in pink).

To investigate divergence of genome sequence, we used four Suessiales species for which multi-isolate genome data are publicly available: two symbiotic symbiodiniacean species (*S. microadriaticum* [3,8] and *Durusdinium trenchii* [9]), the free-living symbiodiniacean species of *E. voratum* [5], and the free-living *Polarella glacialis* [11] that is sister to the Symbiodiniaceae in the order Suessiales (see Methods). Following the approach of González-Pech *et al*. [3], for each pairwise comparison of genome sequences, we calculated the percentage of aligned bases, *Q*, and overall sequence identity of aligned regions, *ID*. Genome sequences from isolates of the same species are highly similar (*Q* > 70.2%, *ID* > 98.6% with minimum alignment length 100 bp; figure 2*a*; see electronic supplementary material, figure S1 for detail), compared to those between species (*Q* < 10.0%, *ID* < 98.6%). High intraspecific sequence similarity was observed despite the diverse geographical origins for isolates from each species (figure 1). Genome sequences of the free-living *P. glacialis* were the most similar (*Q* = 95.5%, *ID* = 98.7%; CCMP1383 against CCMP2088), followed by the symbiotic *D. trenchii* (*Q* *=* 93.3%, *ID* = 99.8; CCMP2556 against SCF082), the free-living *E. voratum* (*Q* = 92.0%, *ID* = 99.4%; RCC1521 against rt-383), and the symbiotic *S. microadriaticum* (*Q* = 78.5%, *ID* *=* 99.7%; CCMP2467 against CassKB8). Among the three *E. voratum* isolates, CCMP421 showed smaller percentage of aligned genome bases against rt-383 (*Q* = 70.2%) and against RCC1521 (*Q* = 79.2%), compared to *Q* = 92.0% observed between RCC1521 and rt383; this is likely to be due to the more fragmented CCMP421 genome assembly, also reflected in the low percentage of mapped sequence reads (electronic supplementary material, table S2). Between the two symbiotic species, the greater divergence observed in *S. microadriaticum* might represent its much earlier emergence and diversification [1]. Alternatively, the lower divergence in *D. trenchii* may be due to the recent whole-genome duplication (WGD) in this lineage [9]. Genome data of multiple isolates from a broader taxon representation of Symbiodiniaceae lineages will help clarify the possible link between intraspecific divergence and facultative lifestyle of these symbionts.

To extend genome comparisons beyond alignable sequence regions, we further assessed sequence divergence using an alignment-free *k*-mer-based approach. This approach was found to be robust against the contiguity of genome assemblies [19], and has been applied successfully to discover distinct phylogenetic signals in different genomic regions of Symbiodiniaceae [5,13]. We followed Lo *et al*. [13] to derive pairwise $D_{2}^{S}$ distances, *d*, based on shared *k*-mer profiles at *k* *=* 23 observed in whole-genome sequences. As shown in figure 2*b*, the lowest sequence divergence was seen in *P. glacialis* (*d* = 0.30), followed by *E. voratum* (*d* = 0.53 between RCC1521 and rt-383; *d* = 0.9 when implicating the more-fragmented CCMP421 assembly), *D. trenchii* (0.54), and the three *S. microadriaticum* isolates (0.72–0.76). This pattern of divergence is consistent with our observations based on *Q* and *ID* in figure 2*a*.

We further assessed the conserved core 23-mers in each species (i.e. *k*-mers common in genomes of all isolates within a species). For each species, we assessed the extent of genome content shared among the isolates based on *x*, the percentage of core 23-mers relative to all distinct 23-mers; in the perfect scenario where genomes of all isolates are identical, *x* = 100%. Using this approach, *E. voratum* and *S. microadriaticum* show similar extent of shared genome content among their corresponding isolates (*x* ranges between 19.5% and 25.2%; electronic supplementary material, table S3). Approximately two-fold greater *x* was observed for *P. glacialis* (52.3–54.9%) and *D. trenchii* (55.6–55.7%); this observation likely reflects the impact of a diploid genome assembly in the former [11] and WGD in the latter [9]. Duplicated genomic regions arising from WGD are resolved over long evolutionary time scales of hundreds of millions of years [20]. Given the recent (approx. 1 Ma) WGD in *D. trenchii*, this species likely has not had sufficient time to resolve genetic redundancy. Regardless, our results here lend support to the general utility of *k*-mer-derived distances in clarifying genome-sequence divergence beyond gene boundaries, which may serve as evidence to guide or complement taxonomic classification of Symbiodiniaceae, and potentially of other dinoflagellates [19].

### 3.2. Intraspecific structural divergence in the genomes of Symbiodiniaceae

To assess intraspecific structural genomic divergence, we identified collinear gene blocks in all possible pairwise genome comparisons for each species; the greater recovery of these blocks and their implicated genes indicates a greater conserved synteny among the isolates in a species. As expected, due to the recent WGD, the two symbiotic *D. trenchii* isolates CCMP2556 and SCF082 displayed the greatest conserved synteny (1613 blocks involving approx. 22% of total genes spanning 181–199 Mbp; electronic supplementary material, table S4). On the other hand, genomes of the symbiotic *S. microadriaticum* (101–196 blocks, 1.9–3.9% of genes, 8.1–17 Mbp) showed less conserved synteny than the free-living *E. voratum* RCC1521 and rt383 (344 blocks, 6.6–8.1% of genes, 51–60 Mbp; electronic supplementary material, table S4); at first glance, this result appears to support observations in an earlier study [3] that the extent of structural rearrangements is greater in genomes of facultative symbionts than those of free-living taxa. However, the greater contiguity of the *E. voratum* assemblies (scaffold N50 length = 720 Kbp for RCC1521, 252 Kbp for rt-383) than that of *S. microadriaticum* assemblies (e.g. scaffold N50 length = 43 Kbp for CassKB8 and 50 Kbp for 04–503SCI.03) represents a systematic bias that would affect recovery of collinear gene blocks. *S. microadriaticum* CCMP2467 (N50 length 9.96 Mbp) (electronic supplementary material, table S1), the sole representation of a chromosome-level assembly, lacks comparative power in this instance. As a case in point, the inclusion of the fragmented assembly of *E. voratum* CCMP421 (N50 length 304 Kbp; 38 022 scaffolds) lowers the extent of conserved synteny identified in *E. voratum* (195–331 blocks, 4.4–7.9% of genes spanning 30–65 Mbp in the CCMP421 genome; electronic supplementary material, table S4), and we identified no collinear gene blocks between the outgroup *P. glacialis* isolates due in part to sparsity of genes on the assembled genome scaffolds [11]. These results in combination suggest that while structural rearrangements contribute to structural divergence of Symbiodiniaceae genomes as postulated in those of facultative symbionts [21] even within the same species, such an analysis based on collinear gene blocks is sensitive to contiguity of assembled genome sequences. An in-depth assessment of structural divergence would require genome assemblies of comparably high quality.

### 3.3. Genetic duplication enables functional innovation

**Figure 3.**
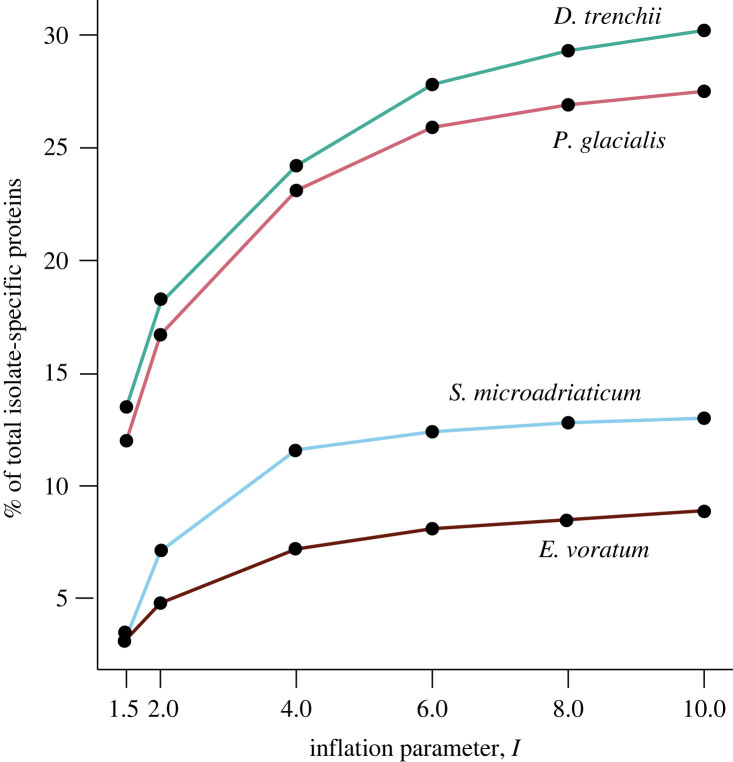
The percentage of isolate-specific proteins in each Suessiales species. Protein sequences were clustered at distinct values of inflation parameter *I* from 1.5 to 10 using OrthoFinder.

Genetic duplication is known to impact genome evolution of dinoflagellates, with genes occurring in high copy numbers implicating essential functions (e.g. [22,23]), possibly facilitated by the introgression of transcripts into the genome following *trans*-splicing of spliced leader in transcription [24,25]. We investigated the evolution of protein families to search for evidence of functional innovation and divergence within species, and its potential connection to lifestyle. For each species, we inferred homologous protein sets with OrthoFinder using sequences predicted from all corresponding isolates; the homologous sets that are specific to an isolate may reflect instances of contrasting divergence in and/or specialization of protein functions (e.g. putative remote homologues), occurring at distinct evolutionary rates. First, we assessed number of isolate-specific proteins for each species based on OrthoFinder results ran at default parameters (i.e. inflation parameter *I* = 1.5). The highest percentage of isolate-specific proteins was observed in *D. trenchii* (13.5% of total proteins), followed by *P. glacialis* (12.0%); these numbers are nearly four-fold greater than that observed in *S. microadriaticum* (3.3%) and *E. voratum* (3.2%; figure 3)*.* To investigate the robustness of this result, we increased the inflation parameter (*I*) for clustering within OrthoFinder that controls the granularity (i.e. higher inflation parameter produces smaller clusters). As expected in all cases, the increase of *I* resulted in an increase of isolate-specific proteins; at *I* = 10, the percentage of these proteins is 30.2% (*D. trenchii*), 27.5% (*P. glacialis*), 13.0% (*S. microadriaticum*) and 8.9% (*E. voratum*). Despite the high synteny and sequence conservation in *D. trenchii*, the substantial number of protein families retained in duplicate after WGD show evidence of isolate-specific divergence and/or specialization in *D. trenchii* where facultative lifestyle has been hypothesized to be the main driver of post-WGD adaptation [9]. In contrast, the comparable extent of isolate-specific protein sets in *P. glacialis* may represent heterozygosity inherent to a diploid representation of the genome assembly [11], distinct from the haploid genome assemblies among the Symbiodiniaceae taxa. None of the *E. voratum* and *S. microadriaticum* isolates showed evidence of WGD (electronic supplementary material, table S5), and thus the similar level of isolate-specific divergence in these species supports the notion of genome reduction in the Suessiales ancestor, with WGD a mechanism for escaping this process to generate functional innovation, as observed in *D. trenchii* [9].

### 3.4. Genomes of free-living species contain a larger number of tandemly duplicated single-exon genes

Tandemly duplicated (TD) genes (i.e. duplicated genes found next to each other on the genome) are thought to facilitate their expression in dinoflagellates [26,27]. Recent studies of whole-genome sequence data [8,11,28] revealed TD gene blocks as part of unidirectional gene clusters. For instance, approximately 40% of the gene repertoire in *P. glacialis* genomes [11] were located in unidirectional gene clusters, many of which encoded functions associated with cold and low-light adaptation. Here we defined a TD block as a block comprising two or more consecutive genes with high sequence identity on a genome scaffold. In our independent survey of TD genes in all 19 available Suessiales genomes, we found the largest number and proportion of TD genes in the free-living lineages of *P. glacialis* (7.8% in CCMP1383, 9.2% in CCMP2088) and *S. natans* (7.1%), followed by the symbiotic *S. tridacnidorum* CCMP2592 (6.5%) and *C. proliferum* SCF055 (6.0%; this taxon was formerly described as *Cladocopium goreaui* SCF055 [29]), with smaller proportions observed in the free-living *E. voratum* (3.9% in rt-383, 4.4% in RCC1521), and the smallest in *S. microadriaticum* (1.0–2.2%) (table 1). Some of the largest TD blocks consisted of 13–16 genes, found in genomes of free-living lineages (*S. natans*, and the *P. glacialis* CCMP1383 and CCMP2088). Among the free-living *E. voratum* isolates, the TD block sizes were slightly smaller, implicating genes encoding ribulose bisphosphate carboxylase (the largest block of nine genes in RCC1521), HECT and RLD domain-containing E3 ubiquitin protein ligase 4 (rt-383, 7 genes), calmodulin (rt-383, 7 genes) and solute carrier family 4 (rt-383, 7 genes) (electronic supplementary material, table S6); these implicated functions are essential for photosynthesis, ion binding and transmembrane transport. However, we cannot dismiss the possibility of genome-assembly contiguity in affecting recovery of TD blocks. For instance, the recovery of TD genes in the chromosome-level assembly of *S. microadriaticum* CCMP2467 is 2.2% versus approximately 1.0% in the other two assemblies, and the recovery of 1.5% in *E. voratum* CCMP421 contrasts to 3.9–4.4% in the other two *E. voratum* genomes. Despite this, a greater extent of TD genes in free-living lineages (*P. glacialis*: 55.2–59.4%; *E. voratum* RCC1521: 23.1% and rt-383: 22.5%; *S. natans*: 21.8%) were single-exon genes, in contrast to the symbiotic *D. trenchii* and *S. microadriaticum* (4.2–9.2%) (table 1). Our results lend support to the notion that tandem duplication may facilitate transcription of genes encoding essential functions implicating single-exon genes, and is potentially more prominent in genomes of free-living taxa than those of symbiotic lineages [11]. Extensive tandem gene duplication has been hypothesized to contribute to longevity and the ease of acclimatization in corals [30]; whether this hypothesis also applies for Symbiodiniaceae remains to be investigated.

**Table 1.** Tandemly duplicated (TD) genes within 19 Suessiales isolates. TD genes were defined as two or more consecutive genes on the same scaffold making up a ‘block', with its size represented by the total number of consecutive TD genes.

species and isolate	number of TD genes	number of TD blocks	median of TD block size	maximum TD block size	number of single-exon genes in the genome	% of single-exon genes among TD genes
*B. minutum* Mf1.05b.01	1225 (3.7%)	569	2	7	2054 (6.3%)	9.9
*Cladocopium* sp. C92	1148 (2.5%)	536	2	8	789 (1.7%)	2.2
*C. proliferum* SCF055	2017 (6.0%)	937	2	7	1870 (5.6%)	9.6
*D. trenchii* CCMP2556	1031 (1.8%)	745	2	6	3828 (6.9%)	9.2
*D. trenchii* SCF082	1045 (2.0%)	645	2	6	5677 (10.6%)	7.5
*E. voratum* CCMP421	495 (1.5%)	233	2	4	1420 (4.4%)	5.1
*E. voratum* RCC1521	1405 (4.4%)	559	3	9	3983 (12.0%)	23.1
*E. voratum* rt-383	1567 (3.9%)	635	3	7	3574 (9.0%)	22.5
*S. linucheae* CCMP2456	737 (2.3%)	348	2	6	255 (0.8%)	8.4
*S. microadriaticum* 04-503SCI.03	437 (1.1%)	206	2	4	2734 (7.1%)	5.9
*S. microadriaticum* CassKB8	418 (1.0%)	200	2	4	3074 (7.2%)	5.7
*S. microadriaticum* CCMP2467	1060 (2.2%)	475	2	7	2770 (5.7%)	4.2
*S. natans* CCMP2548	2499 (7.1%)	1021	2	13	5099 (14.5%)	21.8
*S. necroappetens* CCMP2469	577 (1.6%)	274	2	6	3187 (8.9%)	14.9
*S. pilosum* CCMP2461	496 (2.1%)	236	2	4	1431 (6.1%)	8.3
*S. tridacnidorum* CCMP2592	2491 (6.5%)	1254	2	10	5192 (11.4%)	19.2
*S. tridacnidorum* Sh18	581 (2.3%)	272	2	5	3033 (11.8%)	9
*P. glacialis* CCMP1383	5376 (9.2%)	2095	2	16	15 263 (26.2%)	59.4
*P. glacialis* CCMP2088	4028 (7.8%)	1634	2	14	12 619 (24.4%)	55.2

IE are non-autonomous mobile elements characterized by inverted repeat motifs within introns that are hypothesized to propagate introns into genes [31–33], which have been found to be more prevalent in genomes of free-living dinoflagellate species [5,34,35]. We examined the presence of these elements in the assembled genomes and TD genes for the multi-isolate Suessiales species (electronic supplementary material, table S1). We found the proportion of IE-containing genes overall to be less in Symbiodiniaceae (3.2–6.3%) than *P. glacialis* (10.7–11.5%), a trend also observed in the genome of bloom-forming dinoflagellate species, *Prorocentrum cordatum* (10.4%) [35]. Nonetheless, IEs were only found in a small proportion of TD genes (2.5–5.7%) per Suessiales isolate, suggesting they are neither connected to lifestyle nor play a major role in propagating TD genes in Suessiales (electronic supplementary material, table S1).

### 3.5. Most tandemly duplicated genes undergo purifying selection

**Figure 4.**
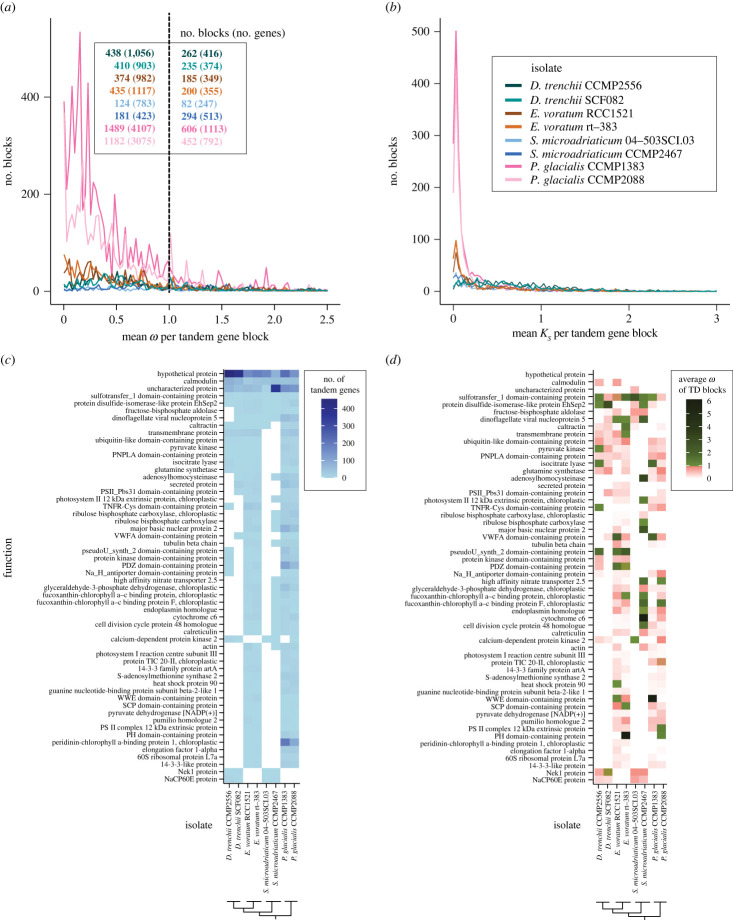
TD genes and their functions in eight Suessiales isolates. The number of TD blocks showing the distribution, respectively, for (*a*) mean *ω* and (*b*) mean *K_s_* of each TD block and its associated TD genes with *ω* < 1 or > 1. Functions encoded by TD blocks that were recovered in genomes of both isolates in one or more species, showing the (*c*) sum of TD genes and (*d*) mean *ω*.

To assess selection acting on TD genes, we focused on the two highest-quality genome assemblies (based on number of scaffolds and N50 length) from each species (i.e. total of eight isolates), excluding the fragmented assemblies of *E. voratum* CCMP421 and *S. microadriaticum* CassKB8. We calculated the ratio *ω* as the non-synonymous substitution rate (*K_a_*) to synonymous substitution rate (*K_s_*) between all possible gene pairs within each TD block (electronic supplementary material, table S6); in general, *ω* > 1.0 indicates positive selection, *ω* = 1.0 indicates neutral selection, whereas *ω* < 1.0 indicates purifying selection [36] among TD genes within a block. Based on this analysis, compared to genomes of symbiotic species, those of free-living species yielded larger proportions of TD blocks with mean *ω* < 1.0, indicating purifying selection, i.e. 71.7% in *P. glacialis* and 67.7% in *E. voratum,* compared to 64.2% in *D. trenchii* and 49.1% in *S. microadriaticum* (figure 4*a*; electronic supplementary material, table S7). In all cases, the mean *K_s_* value per TD block is less than 0.5 (figure 4*b*). The observed mean *ω* values are similar between two isolates of a species (e.g. mean variance of *ω* = 0.26 for both *P. glacialis* isolates; electronic supplementary material, figure S2), suggesting a common pattern of selective pressures acting on TD genes for the species. An exception is the symbiotic *S. microadriaticum* (mean variance of *ω* = 0.16 for 04–503SCI.03 and 0.95 for CCMP2467; electronic supplementary material, figure S2), but more genome data from other multi-isolate symbiotic species will enable the systematic investigation of the possible links between selection acting on TD genes and lifestyles.

To assess functions encoded by TD genes, we focused on TD gene blocks that were recovered in genomes of both isolates in one or more species. Functional annotation of these gene blocks is shown in figure 4*c*, and the mean *ω* value for the corresponding block is shown in figure 4*d*. Genes encoding calmodulin, sulfotransfer domain-containing proteins and disulfide-isomerase proteins were recovered in TD blocks in all eight isolates. Fructose-bisphosphate aldolase, dinoflagellate viral nucleoproteins, and caltractin were recovered in at least 7 of the 8 isolates. Genes in TD blocks recovered only in free-living *P. glacialis* and *E. voratum* encode functions related to photosynthesis (i.e. photosystem I reaction centre subunit III, chloroplast TIC 20-II protein, PS II complex 12 kDa extrinsic protein, and peridinin-chlorophyll *a*-binding protein). In comparison, those in TD blocks found only in the two symbiotic species encode for Nek1 protein that is involved in maintaining centrosomes, and NaCP60E, a sodium channel protein. Most of these functions were encoded by no more than 50 TD genes per isolate (figure 4*c*) in which the mean *ω* per gene block was less than 1 (figure 4*d*). These results do not speak directly to the specificity of gene functions to tandem duplication in the genomes we analysed, given that some gene copies may also occur elsewhere in the genomes. However, our results suggest a tendency for TD genes within a block to undergo purifying selection, regardless of lifestyle.

## 4. Concluding remarks

Our results demonstrate how a facultative lifestyle, or the lack thereof, has shaped the genome evolution of Symbiodiniaceae dinoflagellates. Generation of genetic and functional diversity within species implicates genetic duplication, including tandem duplication of genes. These evolutionary processes are under the constraint of genome reduction that is hypothesized to pre-date the diversification of order Suessiales [5]. Our analysis using whole-genome data uncovered genomic variation and diversity among different isolates or strains within a species, which are otherwise obscured in the identical phylogenetic marker genes they share. Given the small number of strains and species we analysed here, the varying extent of intraspecific genomic divergence of the different lineages remains to be validated using more-extensive whole-genome data that represent greater number of samples per species (e.g. at population scale from more strains and/or locations), and from a broader taxonomic representation. While data generation at such scale remains costly due in part to large genome sizes of dinoflagellates (see [37] for a perspective), our results suggest a potential linkage of facultative lifestyles to intraspecific genomic variations that discriminate free-living and symbiotic species.

## 5. Opening up

Dinoflagellate microalgae from the family Symbiodiniaceae are well known for their role as the ‘solar power plants' of coral reefs. These microalgae allow reefs to flourish in nutrient-poor tropical waters via provision of fixed carbon through photosynthesis, as well as essential nutrients. Breakdown of the coral–alga symbiosis (i.e. coral bleaching) due to environmental stress puts corals at risk of starvation, disease and eventual death. Much effort is being expended to understand the basis of the coral–alga symbiosis to enhance coral resistance to thermal stress. Genome data from these microalgae provide a valuable resource to achieve this goal. Earlier research has revealed extensive sequence and structural divergence among distinct species and genera of Symbiodiniaceae. This study investigates genome divergence of Symbiodiniaceae at a finer resolution, specifically in comparing genomes of multiple isolates from different species. Results from this work demonstrate the remarkable genomic divergence among Symbiodiniaceae taxa even among isolates. These data underline the importance of considering intraspecific divergence that is driven by local adaptation, and argue against the one-size-fits-all approach when designing a robust coral–alga symbiosis.

## Data Availability

The key data used in this study were published genome data available from the cited sources with the following detail for which GenBank accessions are noted where applicable: *Symbiodinium microadriaticum* CCMP2467 [8] (GSE152150), CassKB8 [3] (GCA_905221625) and 04-503SCI.03 [3] (GCA_905231925); *Durusdinium trenchii* CCMP2556 and SCF082 [9] (https://doi.org/10.48610/27da3e7) [38]; *Effrenium voratum* RCC1521 (GCA_963377175), rt-383 (GCA_963377275) and CCMP421 (GCA_963377065) [5] (https://doi.org/10.48610/1f0377a) [39]; and *Polarella glacialis* CCMP1383 [11] (GCA_905237085) and CCMP2088 [11] (GCA_905237095). Sources of other genome data are detailed in electronic supplementary material, table S1. Supplementary material is available online [40].
